# Extracts and Gleanings from Our Exchanges

**Published:** 1873-12

**Authors:** 


### PART II.

### EXTRACTS AND GLEANINGS FROM OUR EXCHANGES.

### *MULTUM IN PARVO.*

A Good Liniment.—This liniment is good for man or horse, to be used indiscriminately when a liniment is is required. This is the most penetrating, soothing liniment made. To this ℞. I add com. liq. ammonia, laudanum or chloroform to suit each case. I do not generally add anything. It is good enough "straight." I know no name for it save "Mitchell's Liniment."

℞. Oil origanum, oil wormwood, gum camphor, aa ℥ ss; oil sasafras, ʒ ij.; spirits turpentine, ℥ ij.; alcohol, 95 per cent., pint. i.

Mix and apply every 3 hours thoroughly with the hand.

For neuralgia, add chloroform and tinct. opii., q. s.; for rheumatism, add com. liq. ammonia, q. s.

Cost of the above quantity is less than 75cts. I sell it at 25cts. per oz.; profit $4.25.

Dr. Cameron's Domestic Remedy for Dropsy.—As improved by myself. Where a tonic and special diuretic is wanted, I know no equal. I say this after thorough test and actual experience in my practice with many cases with entire satisfaction. Two of the cases I appointed a time when I would "tap them," but our remedy was so happy I did not get a chance to display my skill.

℞. Fresh mullen leaves, ℥ ij.; fresh cedar leaves, ℥ ij.; pure nitrate potash, ʒ ij.; best Holland gin, pt.i. Mix.

Dose. One tablespoonful 3 times a day, one-half hour after eating. (The leaves must be cut up in small pieces.)

Thos. S. Mitchell, M. D.

*Hamilton, Ga.*, Nov. 17, 1873.

The Internal Use of Aconite in Neuralgia.—A. Leslie Mease, M. B., of Armagh, Ireland, writes as follows to the editor of the London *Medical Times and Gazette:*

I send you a few notes of cases showing the great use of tincture of aconite, both alone and in combination with other remedies, in neuralgia and muscular rheumatism. I now speak of its *internal* use, in contradistinction to its *external.* I give five cases, each the representative of a different class of the same affection:

Case 1.—Mrs. D—— had been for some time under my care for violent paroxysmal neuralgia of the face, partially due to carious teeth, and partially to gastric derangement. As she would not have her bad teeth extracted, nor attend to her digession or bowels, I was perpetually trying to invent something new to mitigate the agony she suffered. On August 29 she began to take tinct. aconit. *m*x., tinct. colchici *m*iij., brom. potass. gr. v., each third hour, with spt. chloroform *m*xx. Having been prepared for this treatment by a purgative, on the 30th, she had almost, and on the 31st completely recovered; and this happy state of things lasted for six weeks. Of course, in this case, extraction of the bad teeth and proper attention to the digestive organs, would have afforded most hope of a radical cure.

Case 2.—Mrs. L. M——, in fourth month of pregnancy. Headache, vomiting, very violent pain in back and legs, producing horribly distressing twitchings of the latter. After a dose of magnesia (effervescent citrate), she began to take the following each third hour: Bicarb. Potass. gr. v., brom. potass. gr. ij., tinct. aconit. *m*v., acid. hydrocyan. dil. *m*iij., spt. chloroform *m*xx. In about an hour the pain and twitching began to diminish, and in nine hours only stiffness and soreness of the muscles remained to remind her of what she had suffered.

Case 3.—Miss F——, a hysterical subject, with facial neuralgia, coming on at night and totally preventing sleep. No carious teeth. Tinct. aconit. *m*x., brom. ammon, gr. x., spt. chloroform *m*xl., was found to give a good night, attention, first of all, having been paid to the state of the bowels.

Case 4.—Mr. S—— neuralgia testis, not very severe, but accompanied by a tendency to spermatorrhœa. The latter got better under treatment, but as the pain continued, I ordered the following: Tinct. perchlor. ferri *m*xij., tinct. aconit. *m*iv., spt. chloroform, *m*xv., twice daily, with infusion of quassia and a pill at night, containg camphor gr. ij., ext. hyoscyami gr. ij., pulv. aconit. gr. ss. This was efficacious in removing the pain in about ten days; but I may remark that even this amount of aconite produced so much tingling in this patient that he was constrained to take the mixture but once daily.

Case 5.—I myself, for years, have suffered, off and on, from severe muscular pains, in the lower extremities in particular, and I rarely ever find that two doses of tinct. aconit., from *m*v. to *m*viij in each, at an interval of two hours, fail to give complete relief.

I have now given five cases, each a representative of a different class of rhenmatic and neuralgic affections, in which I have found the internal use of tincture of aconite exceedingly useful. It remains for me to say in what class of cases I have not found such good effects. They are two—first, in acute rheumatism attacking the joints; and, secondly, in lumbago, its internal use has proved of much less value. In the latter affection, however, complete relief may often be obtained by the application of chloroform, liniment. aconit., and liniment. belladonæ, in equal parts, on lint, with oiled silk over it.

In conclusion, I would add that constipation, or a disordered state of the stomach, or both, should be remedied before commencing the use of aconite, or *at least*, in such cases, suitable remedies for the relief of such a state of thing should be combined with it. As a rule, aconite seems to have more effect when combined with spirit of chloroform than when taken alone—at least as far as my experience goes.

It is well to commence with a small dose, as I know of no remedy so variable in its effects on different constitutions. I have seen a single dose of eight minims produce decided tingling *all over the body* in an adult, and I have given as much as twenty minims each third or fourth hour without any tingling at all.—*Nashville Journal of Medicine and Surgery.*

Trismus Nascentium.—Dr. G. T. Maxwell, (Phil. Med. Times,) in one successful case, gave the following treatment:

℞. Chloral. hydrat., gr. vj.; syrup. simp.; aquæ, aa ℥j.

I directed one-fourth to be administered every two hours. The effect of the first two doses was to produce sound and seemingly natural sleep, during which the spasms were suspended. On awaking, the spasms recurred; but it was thought they were less violent. In the evening I directed the chloral to be given often enough, and only when required, to keep her asleep all night. The next morning the amelioration of her symptoms was quite perceptible; she nursed with greater ease; and when, after prolonged intervals, the spasms returned, they were manifestly less rigid. The effect of the chloral was maintained throughout that day and the next night, with steady progress of the case towards recovery, the intervals between the doses being increased in length as the disease diminished in severity; and after the third day all treatment was discontinued without a recurrence of the trismus.

How to Clean Drainage Tubes without Removing them.—M. Dr. Lesueus describes a very simple method of his adoption by which debris can be removed from drainage tubes without removal. This consists in attaching a pellet of cotton or charpie to a long wire, double the length of the tube. The wire is inserted into the tube before its adaptation and then protruded so that the cotton no longer lies in its calibre. The simple partial withdrawal or further insertion of the wire runs the cotton plug through the tube and effectually cleans it.—*Clinic,* Nov. 30, '72.

TREATMENT OF DISEASE IN CHILDREN.—By Eustace Smith, M. D., Lond., Physician to His Majesty the King of the Belgians.

There is one class of remedies which is of singular value in the treatment of the diseases of young children—viz: the alkalies. In all children (in infants especially) there is constant tendency to an acid fermentation of their food. This arises partly from the nature of their diet, into which milk and farinaceous matters enter so largely; partly from the peculiar activity of their mucous glands, which pour out an alkaline secretion in such large quantities. An excess of farinaceous food, therefore, soon begins to ferment, and an acid is generated, which stimulates the mucous membrane to further secretion. In all chronic diseases, and in many of the acute disorders, this sour condition of the stomach and bowela is present. Alkalies are therefore useful—firstly, in neutralizing the acid products of this fermentation; and secondly, in checking the too abundant secretion from the mucous glands. A few grains of soda or potash, given an hour or two after taking food, will quickly remedy this derangement and remove the distressing symptoms which arise from it. In the chronic diseases, indeed, attention to this point is of especial importance; tor by placing the stomach and bowels in a healthy state, and insuring a proper digestion of food, we put the child in a fair way of recovery, and prepare the way for the administration of tonic and strengthening medicines, by which his restoration to health is to be brought about.

In prescribing for infants, an aromatic should always be included in the mixture. The aromatics are useful, not only for their flavoring properties, but also for their value in all those cases of abdominal derangement where flatulence, pain, and spasm, resulting from vitiated secretions and undigested food, are present to increase the discomfort of the patient. Such dyspeptic phenomena are usually relieved rapidly by the use of these agents; and aniseed, cinnamon, caraway-seed, or even tincture of capsicum in minute doses, will be found important additions to the prescription in all cases where alkalies are required.

In prescribing for children, the proper dose of a medicine cannot always be calculated according to the age of the child, and does not in all cases bear the same proportion to the quantity suitable for an adult. For certain drugs children show a remarkable tolerance, while to the action of others they show as remarkable a susceptibility. Thus, opium, it is well known, acts upon a child more powerfully than would be expected, judging from the mere difference of age. It should therefore be given to infants with a certain caution, especially if the child be enfeebled by disease.

It is, however, a medicine which is of especial value in the treatment of the diseases of infancy, and may be given without fear if care be taken not to repeat the dose too frequently. Belladonna, on the contrary, can be taken by children in large quantities. A child of two or three years old will bear without inconvenience a dose which in an adult might produce very uncomfortable symptoms. Lobelia, again, is a remedy which is very well borne by children.

Dr. Ringer has given it to "very young children" in doses of five minims every hour, and in no case has he noticed any ill effects to follow its administration. Arsenic should be given to children over five years of age in the same dose as that used to adults, and infants of a month or two old will take one drop of Fowler's solution three times a day with great benefit in cases of gastric catarrh.

The influence of mercury upon young children deserves remark. It seldom in them produces stomatitis or salivation; but an excess of the drug is not therefore harmless; its influence is seen in the irritation of the alimentary canal which it so often excites, and in the profound anæmia which it induces. The anæmia which is so common a sequence of constitutional syphilis in infants is no doubt often a result of too long-continued mercurial treatment.—*Medical Times & Gazette, April, 1873.*

Diarrhœa in Teething.—In a clinical lecture "On the Primary Dentition of Children," by Francis Minot, M. D., Harvard (*Boston Medical and Surgical Journal,* January 2, 1873), in speaking of the diarrhœa complicating teething during hot weather, recommends the common chalk mixture with the addition of one-fourth part of tincture of kino, which increases its astringency, and also keeps it from turning sour in hot weather. If the diarrhœa be not checked by this mixture, one drop of laudanum may be added to a dose, but not oftener than three times a day, in children under two years old. Diarrhœa is most apt to attack children who are brought up on the bottle; hence, if the case be urgent and do not yield to treatment, a wet-nurse should be procured if possible. When this cannot be done, he would strongly recommend the method of preparing milk with arrow-root and gelatine, found in the treatise on "Diseases of Children," by Drs. Meigs and Pepper. Brandy is very useful to a teething child exhausted by diarrhœa, which should be given once in three or four hours, or oftener in urgent cases. The dose is ordinarily from five to twenty-five drops, given in milk; but if there be much prostration the physician need not fear to increase the amount.

Poisonous Doses of Chloral.—The smallest dose which has produced poisoning is 2½ scruples, while 460 grains have been taken without unpleasant consequences. The symptoms are diminished frequency of respiration, redness of the conjunctiva, contraction of the pupils, lividity of the the lips, and falling of the jaw. The character of the pulse has not been found to be uniform. The treatment should consist in: 1. Removing the poisonous matter or diluting it by water, tea, coffee, or other fluids; and 2. Restoring respiration. Too much reliance must not be put upon so-called antidotes, such as strychnine, physostigmine, morphine, camphorated ether and ammonia. These remedies cannot always be trusted. Possibly transfusion of blood, which has been successful in cases of poisoning by chloroform, may also prove useful in these cases.—*Med. Chir. Rundschau,* Oct., 1871.

DIPHTHERIA.—Dr. V. J. Fourgeaud, (Western Lancet) writes: "I have relied principally on the liquor ferri subsulphatis, both for local application and internal administration, considering it the most valuable remedy we possess for that class of diseases characterized by exudations, vegetations, false membranes and abnormal formations and growths of a humid and superficial nature on the mucous membranes.

I apply the undiluted solution to the affected parts by means of a camel's hair brush or, better, of a piece of sponge, smaller than is generally used for throat probangs, attached to a suitable holder.—The sponge should be well moistened with the solution, but should be pressed a little before using, to free it from an excess of fluid. In applying the medicated sponge, it should be gently rubbed on the affected parts. The peculiar action of the solution on the exudations or false membranes, will cause them to break up more or less completely, according to their different degrees of adhesiveness to the mucous membranes, as shown by the pieces adhering to the sponge, and, also, by their presence in the immediate expectoration. After each application (I make from two to four in the twenty-four hours, according to the exigency of the case) a mouth-full of water, as a gargle or swallowed, will relieve a certain degree of constriction which follows the use of the astringent. Far from complaining of pain, the patients experience decided relief after the use of the lotion. I consider this application by far the best I have ever used. It may be employed much more freely and with less danger than violent caustics, such as muriatic acid, nitrate of silver, etc., commonly resorted to. I know of no other which will break down so effectually all exudations, vegetations, pultaceous concretions, as well as the characteristic false membranes of diphtheria, whether they are situated in the mouth, pharynx, or in the vagina, neck of the womb, or any other accessible parts of the mucous membrane. I have used it with gratifying success in many cases of sore throat, in scarlet fever, in stomatitis and aphthous affections of children. Three or four applications will generally free the buccal and pharyngeal cavity of all vegetative or poultaceous growths or coatings. But when the disease takes the form of true diphtheria, when the false membranes are well characterized, although the progress towards cure is generally evident after each application, it is, of course, slower. In these cases the medicated sponge should be applied every six hours, until the diseased surface becomes entirely free from false membranes. The sponge should be well cleaned after use.

With the local treatment, I prescribe the following for internal administration:

℞—Potassæ chloratis, ℈ii; Glycerinæ, ℥i; Quiniæ sulphatis, ℈i; Liquoris ferri subsulphatis, gtt xx; Aquæ, ℥iii; M.

Half a teaspoonful to a tablespoonful of this mixture, according to age, to be taken every four hours.

I have found the small quantity of the liquor ferri subsulphatis, in the above recipe sufficient for full remedial action on the system. The quantities of the subsulphate and quinine may undoubtedly be increased. However, I hold the following a good maxim in practice: Be sure to do enough, but be doubly sure not to do too much. It dissolves the given proportion of quinine perfectly, and the mixture is of a beautiful color, clear and free from precipitate.

Besides being a good tonic, it has a decided effect on the morbid secretions of the respiratory tubes, and of the stomach and digestive canal. I have found it an excellent adjuvant in the treatment of croup and of many forms of low fevers attended with gastro-enteric debility, thickly coated tongue, and curd-like and other exudations or poultaceous patches of the mucous membrane.

While administering this internal treatment in connection with the local, strict attention should be paid to cleanliness, pure air and warm clothing, and the patient should take abundantly of milk, beef tea, plain nourishing food, and a stimulating beverage, such as brandy and water."

Medicine for Children.—In prescribing for children, the proper dose of a medicine cannot always be calculated according to the age of the child, and does not, in all cases, bear the same proportion to the quantity suitable for an adult. For certain drugs, children show a remarkable tolerance, while to the action of others they show as remarkable a susceptibility. Thus, opium, it is well known, acts upon a child more powerfully than would be expected, judging from the mere difference of age. It should, therefore, be given to infants with a certain caution, especially if the child be enfeebled by disease. It is, however, a medicine which is of especial value in the treatment of the disease of infancy, and may be given without fear if care be taken not to repeat the dose too frequently. Belladonna, on the contrary, can be taken by children in large quantities. A child two or three years old, will bear without inconvenience a dose which in an adult might produce very uncomfortable symptoms. Lobelia, again, is a remedy which is very well borne by children. Dr. Ringer has given it to "very young children" in doses of five minims every hour, and in no case has he noticed any ill effects to follow its administration. Arsenic should be given to children over five years old in the same dose as that used to adults, and infants of a month or two old will take one drop of Fowler's solution, three times a day with great benefit in cases of gastric catarrh. The influence of mercury upon young children deserves remark. It seldom, in them, produces stomatitis or salivation; but an excess of the drug is not, therefore, harmless; its influence is seen in the irritation of the alimentary canal, which it so often excites, and in the profound anæmia which it induces. The anæmia which is so common a sequence of constitutional syphilis in infants is no doubt often a result of too long continued mercurial treatment.—Medical Times and Gazette, April 12, 1873.—Dr. Eustace Smith of London.

Neausea of Pregnancy.—Dr. T. K. Bailey recommends the following: ℞. Oxalate of Cerium, grs.viij; Sub. nit. bismuth, grs. ix. M. f. pulv. No 12. S. One, two or three times daily.

ANODYNES AND ANÆSTHETICS IN OBSTETRICS.—At a recent meeting of a medical society at Ithaca, Dr. S. P. Sackett read a paper on "Progress in Obstetrics and Surgery," from which we take a passage or two from the Medical and Surgical Reporter :

"In common with many others, I now believe that we are able to do more to mitigate the pains of child-bearing with the use of medicine than was formerly done. During the past ten years I have given belladonna in small doses daily, to pregnant women about two weeks previous to their confinement, and have observed its effects upon about 75 to 100 cases annually. I believe that it facilitates labor, perhaps, because it relaxes or renders inert the circular muscular fibres of the uterus. It should not be given too freely, as I think that it has had the effect in some cases of prolonging the period of utero-gestation.

"During labor medicines are given to relieve pain more than formerly. Some use chloral, though I am not aware that its use is on the increase. One physician has suggested to me that it had the effect to stop flowing; it will be well for us to note carefully any such effect. The use of anæsthetics in obstetrics is probably not much on the increase; but those that have used them seem to be generally satisfied with the effect. Whisky and chloroform given together internally are used and recommended, to what extent I am not prepared to say.

"My own observation leads me to prefer morphine to any other agent for ameliorating the sufferings of labor. I am aware that it may justly be said to retard, in many cases, the progress of labor, but my observation leads me to conclude that in very many cases it may be given to the extent of evident relief to pain, without in any degree delaying the final favorable termination. Some have said that it makes the pains more efficient; perhaps it does where there is a rigid os; but it may be given with advantage in other cases. If I see a case but little advanced, and advancing quite slowly, so that it is likely to last some twelve to fifteen hours, I would always give a dose of morphine, perhaps large enough to produce sleep; there would still be the same slow progress in the case, and finally a few efficient pains at the last would effect delivery at the time when it was likely to occur without the opiate, but the woman would suffer less and be less exhausted. I sometimes give morphine, one or two $\frac{1}{8}$-gr. doses, where the pains are severe, near the termination of labor, rather with the design of a little present relief, and also of having the patient comfortable after the child is born. We should be not only anxious to deliver the woman, but also should be careful, if the constitution is weak, that the *shock* is not too great for the woman to bear.

"Of the hypodermic injection of morphia, I can say but little from observation, but I think that hereafter it will be frequently used. For example, in some cases a desirable state of quietude of the uterus was induced where the liquor amnii had passed, and it was desirable and necessary to turn and deliver the child."—*The Doctor.*

The Use of Ergot.—Dr. T. K. Spendee writes (*British Medical Journal*) as follows: "I have given ergot in some cases of neuralgia, according to the advice of Dr. Woakes, of Luton; but, though I have had particularly good results, I have not been able to remove pain entirely by the use of ergot alone. I can endorse all the favorable views of ergot in the treatment of hæmoptysis, as related by Dr. Dobell and Dr. Anstie. I have used the medicine for this purpose during several years past, having been originally led to do so by a consideration of its therapeutic analogies. It does not yet seem to be clearly defined whether there is any stage of phthisis, even the most advanced, which is absolutely beyond the control of ergot, when spitting of blood occurs. Of the exceeding value of the medicine in these cases (though it now and then unaccountably fails), there can be no doubt whatever; and, as the facts are very little known, attention cannot be too often called to them. The action of ergot on the uterus is a proverb; why should it not affect in a similar way a neighboring organ—the bladder? I have found that that quasi-paralytic condition of the bladder, which may come on in middle-aged persons from over-fatigue or from simple want of power in the coats of the organ, is greatly relieved by the continuous use of ergot, and may be altogether removed. The so-called hysterical paralysis of the bladder in young women is admirably treated with the same medicine (though I cannot deny the occasional necessity for the use of the catheter). Whether this want of power be simply motor weakness, or secondary to some variety of abdominal neuralgia, there is no more splendid combination of medicines than ergot and strychnia (half a drachm of the fluid extract of ergot and five or six minims of the *liquor strychniæ*, Ph. B., in chloroform-water, three times a day); and these doses should be continued perseveringly for several weeks, as a very rapid benefit cannot be expected.

Fluid Adhesive Plaster—The following formula for emplastrum adhæsivum fluidum, given by Mr. Enz, of Sembach, offers some interesting features. Take of dammar resin, finely powdered, 560 parts; oil of almonds, 142 parts; melt till the mass flows smoothly, and when half cold, add, by degrees, 225 to 240 parts of spirit ether, in which aniline, free from arsenic, or any other coloring matter, has been dissolved. The plaster thus obtained is of the consistence of a balsam. The dammar resin is easily soluble in fat oils; by the addition of spirit of ether it is partly precipitated, but in a very finely divided, doughy state. Dammaryl and hydrate of dammaryl are not soluble in alcohol, and impart to the mixture an extraordinary sticking power. This plaster can be directly applied to wounds, and then dressed with cotton or linen, or spread thereon by means of a brush. What renders this plaster especially useful is, its easy miscibility with other ingredients; for example, acid carbolicum purum, hydrarg. chlorat. corrosiv., morphium and its salts, iodide of potash, which are all soluble in alcohol; further, with powdered cantharides, belladonna, hemlock, etc.—*Medical and Surgical Reporter.*

CROUP AND DIPHTHERIA.—Dr. Schultz (in the *Weiner Med. Wochenschrift*) recommends the inhalatiou of bromine in a strong solution of brom. potass. as a prompt, safe, and speedily beneficial means of treating these formidable diseases. His formula is bromine and bromine potassium, each three-tenths of one gramme (4½ gains) to water 150 grammes (about ℥ iv ss.) A sponge is soaked in the solution and placed in a funnel of stiff paper, and held over the nose and mouth for inhalation (just as is done in the inhalation of chloroform), the inhalation being continued for five or ten minutes, and repeated every half hour or hour. This preparation is decomposed by light, and should be kept in glass-stopped vials, away from the light. To use it with the atomizer would no doubt be preferable, as by this means the minute particles would follow the air in every direction into the air tubes and over the epiglottis, trachea, etc. The bromide of ammonia, used in this way, was long ago recommended by Dr. Youart, of Indianapolis.

In the *Medical Times* is an extract from the *Gazette Hebdomadaire* of a paper by Dr. Daquillon, who recommends, when symptoms of impending suffocation arise in membraneous croup, that a sponge be well attached to a whalebone in the form of a probang. The sponge is to be dipped in the pure liquor ammonia and thoroughly squeezed, and then passed down the child's throat, which is made to inhale the vapor of ammonia as long as it possibly can. An abundant secretion is soon established from the respiratory mucous membrane, and an immediate detachment and expectoratiou of the false membrane, and along with this a marked diminution of the dyspnœa, cough, and other alarming symptoms. Dr. Daquillon assures us this treatment has frequently saved the lives of children whose case, a few moments before, appeared next to hopeless.

Dr. Coates, of Otsego, Michigan, says that he once encountered an epidemic of croup and diphtheria mixed, which was very fatal. He, however, succeeded in curing his cases by using frequently through the day, with the atomizer, the following:

℞—Bromide potass. ʒ ij.; comp. sol. of iodine ℥ ij.; water ℥ iv. He says that the false membranes *melt away* under its action, and the bad symptoms subside rapidly. He mentioned the case of an infant, a year old, in the last gasps of life, from acute membraneous croup, that was saved in ten minutes by the above. The infant, he believed, would have died in ten or fifteen minutes.—*Medical Archives.*

EARTH AS A SURGICAL DRESSING.—Dr. Alex. Rattray, (London Lancet) used this form of dressing on an obstinate ulcer of the matrix of the great-toe nail, which resisted every remedy, but soon healed under poultices of common earth. Under the same application the cicatrisation of ordinary ulcers, wounds, boils, etc., was equally favorable and rapid, as was the resolution of erythema of the skin. The dried and finely sifted earth was used either in the form of poultice or thinly spread ointment-wise on calico, or simply sprinkled over the sore.

Cerebro Spinal Meningitis.—Prof. W. R. Bowling, of Nashville, (Nashville Journal of Medicine and Surgery,) in a discussion of the treatment etc., of this disease by the members of the State Medical Society, said :

"Sulphate of morphine, easily divided and made palatable by a convenient menstrum, is my favorite. I order one grain of the sulphate of morphine to be rubbed up with an ounce of lemon syrup. A teaspoonful of this in ice water, may be given to a child of from six to ten years of age as often as is necessary to keep him from raving. We begin every case with it. A child one year old would take two or three drops, or the 240th part of a grain, as often as necessary. I have a number of patients from twenty to thirty months old ; these take five drops, or the 120th part of a grain of the sulphate, every five hours. Once in three or four days a little warm water is thrown up the bowels. They drink all the milk I can get them to drink, and they have ice-water *ad libitum.*

We are engaged, with the assistance of several able practitioners of the city, to secure an indication for the repetition of the morphine, from the size of the pupil, but have progressed but little.

The after effects of the morphine are often unpleasant, when chloral may be satisfactorily substituted. But the quieting effects of chloral most generally ensue eight hours after its administration, and not before, and this should be remembered. We give it in ten-grain doses, dissolved in water, to patients from eight years of age and upward, and in less doses to smaller children, and repeat according to circumstances.

Twenty or thirty drops of McMunn's elixir of opium, with a tablespoonful of the watery solution of assafœtida in a little warm water, thrown up the bowels at bed-time, in patients from eight to fifteen years of age, "acts like a charm."

Wine is often valuable, and food must not be neglected. Cold cloths to the head, and inhalation of ether are the best remedies for the distracting pains.

Very simple cure for a formidable disease, you may say—so were the five smooth stones that sent Goliah to his long home, and saved Israel ; or the dippings in the Jordan, that made the unbeliever whole. Dr. Daniel Caldwell has left in his autobiography this remarkable saying : "The greatest physicians I have known were remarkable for the simplicity of their remedies, and the fewness of them."

Dressings for Ulcers.—Dr. Ohleyer advocates the use of magnesia, which he has found very successful in the dressing of certain ulcers when fermentive processes retarded healing. Magnesia neutralizes the acids present, prevents the access of oxygen to the surface, and protects the granulations. The author especially applies it to atonic ulcers ; cases in which the skin is without epidermis and in which there is danger of suppuration ; inflamed and painful sores ; wounds which require to be stimulated or to be withdrawn from the influence of air, or in which suppuration should be diminished or modified, and in erysipelas of the face.

On Endometritis.—At a meeting of the Dublin Obstetrical Society, December, 1872, Dr. Lombe Atthill read a paper on this affection:—

It might be defined as a low inflamation of the uterine mucous membrane, with vascular engorgement and implication of the grandular structure of the organ. Sometimes the cervix was engaged. The symptoms of the endometritis were pain, leucorrhœa, dysmenorrhœa, menorrhagia, and reflex irritation. Pain was generally referred to one or all of three localities, viz., to the sacrum, to the edge of the false ribs, thence shooting to the shoulder on the left side, and to a point just over the pubes. The second was often almost pathognomonic of the disease. The physical signs of endometritis were, increased length of the uterine cavity, increased size of the same, increased bulk of the whole fundus, augmented sensibility of the uterine mucous membrane, a patulous *os internum*, and often an abnormal sensitiveness of the mucous membrane. In the treatment, palliative measures, including rest, warm hip-baths, mild aperients, and, above all, local depletion, sometimes acted beneficially. The last named might be affected by leeching, but was far more effectually carried out by puncture of the cervix in one or two places to the depth of an eighth of an inch or thereabouts. Dr. Atthill exhibited a knife designed for this purpose. In severe cases operative interference was necessary; either by injecting fluids into the cavity of the uterus, or by passing up a piece of solid caustic, or by the application of fuming nitric acid, the acid nitrate of mercury, or other active agent. Dr. Atthill considered the application of fuming nitric acid to the interior of the uterus as simple, safe, and painless; and to Dr. Kidd belonged the priority of the adoption in Ireland of the internal application of the acid; while in America Drs. Miller and Marion Sims had previously carried out the same practice. The author advised the preliminary dilatation of the cervix uteri with sea-tangle or sponge-tent. The anterior lip of the uterus was then seized with a hook, and a stilette armed with a comparatively thick layer of cotton or roll of lint was passed rapidly up to the fundus. Strong nitric acid thus applied seldom caused any pain, and was not followed by any grave consequences, as the injection of even weak caustic solution often was. In all cases where it was healthy, the cervix uteri should be protected from the action of the nitric acid. To reach all parts of the uterine cavity with the acid, the author had devised an intra-uterine speculum, which could be expanded by means of a screw working through a long handle. The details of three cases of endometritis were given, and Dr. Atthill concluded by a vindication of the method of cauterization of the uterus with nitric acid from the objections raised against it.

Glycerite of Lime for Burns.—This is prepared from recently slaked lime, one part; glycerine, fifty parts; Chlorinated hydrochleric ether, one part, and is said to soothe the pain and to diminish the inflammation.

New Use of Barnes' Dilators.—Mr. H. M. Morgan records (*British Medical Journal*) two cases in which he resorted to Barnes' dilators with advantage. The first was a case in which the waters broke early, and the os small and unyielding. Mr. M. thought that by making an artificial bag of waters, he would materially assist labor, so with some difficulty he managed to introduce Barnes' largest bag within the os by means of an uterine sound. When once it was there, it was easy to pump in nearly a pint of cold water; and the labor then progressed very well, each pain dilating the os by means of the artificial bag, quite in a natural way.

The second case was one of miscarriage at the eighth month, with profuse hemorrhage, placenta prævia and unyielding os. Mr. M. ruptured the membranes with a stilette, and then by means of a long pair of ovum forceps, he passed Barnes' largest bag—rolled up small—quite into the womb, and afterwards pumped into it nearly a pint of warm water. As she had no pain worth mentioning, he had given her forty minims of liquid extract of ergot before puncturing the membranes. As soon as he had filled the bag with water he commenced dilating the os himself by drawing at the tube till his finger and thumb could reach the root of the tube in the vagina. The result was that in little more than five minutes, he pulled a soft bag, as large as an ordinary new born child's head, through the os; and then the vagina and perineum were gradually dilated in the same way until the bag came right away. He found the head presenting, and pains were coming on moderately; but as there was no time to be lost, he preferred not to wait for nature to act; so with one hand internally and the other externally, he turned the child and brought down a foot, and soon completed the labor with the aid of the patients own pains and efforts. The placenta was expelled naturally, and the womb contracted well after it. Dilation, he says, would not possibly have been accomplished so quickly, so easily, so painlessly and so safely by the hand. Moreover, this bag being pressed against the bleeding placental vessels in its passage through the os, compressed them and checked the hemorrhage in the same way as the head does in those cases where the pains are strong enough to keep it well pressed against the os.—*Am. Jour. Med. Sciences.*

Melancholia.—Dr. Hostermann claims to have obtained successful results in the treatment of simple melancholia with nitrite of amyl, given in inhalations from twice to four times a day, in doses of from four to five drops inhaled for about forty seconds. He finds that this agent has a notable effect in increasing the quickness of the pulse. It also widens the calibre of the capillaries in the skin and in the head.

Dr. Otto Obermeier employs æthyl alcohol in the same form of insanity. By this name he appears to designate a fluid composed of 30 per cent. of rectified spirit mixed with water, "with aromatic additions." With this compound he has obtained much success in cases of melancholia, and never noticed any bad symptoms.

On the Treatment of Gonorrhœa by Medicated Bougies.—From a paper in a recent number of the Annal. de Dermatol. et de Syphil., we find that Dr. G. Dorey, late of the Hospital du Midi, has been extending the experiments of the late Dr. Liegeois, who introduced to the notice of the profession in France, the employment of *bougies Reynal* in the treatment of gonorrhœa and analogous diseases.

Dr. Lorey's experiments were tried on some 80 cases of gonorrhœa, in 60 of which the disease had been but recently contracted, while in the remaining 20 it was more or less long-standing, and in one case even six years had elapsed since the disease had first made its appearance.

Although these latter cases had all of them resisted ordinary medical treatment, yet Dr. Lorey found no difficulty in curing even the most chronic case, and this in the comparatively short space of three weeks. The author's success was not so wonderful with the acute cases, yet it was such as to warrant him in strongly recommending the use of the bougies Reynal in these cases.

The bougies of Reynal which are used by Dr. Lorey are composed of a central firm rod of gelatine, on which is spread a mixture of gum and the appropriate medical agent, such as sulphate of zinc, tannin, belladonna, opium, &c. The quantity of opium or zinc used in each bougie is $\frac{3}{4}$ of a grain. The bougies should be equal in length to the urethra, and in diameter to a No. 4 catheter.

Before using, it should be plunged into cold water, after which its introduction is perfectly easy and free from pain, no matter how irritable the urethra may be.

The bougies are used once each day, and are kept in the urethra for an hour and a-half at each application.

The author uses belladonna or opium on the bougies in the earlier stages of gonorrhœa, and congratulates himself on the results which are as follows:

1st. Little or no pain on micturition.

2nd. Nocturnal erections are no longer to be dreaded, as he recommends the use of the bougie before going to bed.

These bougies are indicated in the first stage of gonorrhœa, to soothe the insupportable pain which follows or accompanies the act of micturition. In this case they have a double action, soothing pain, and at the same separating the inflamed walls of the tube.

In the second stage of gonorrhœa, and in chronic cases, the bougie of belladonna and sulphate of zinc is indicated, and its efficacy is very great.

Dr. Lorey thinks that no therapeutic agent at present in use can lay claim to anything like the results which have followed the use of this treatment, and adds that these results are effected as much by the mechanical agents with which they are covered.

We must not attribute these results to the sanitary conditions of the patients in the Hospital du Midi, nor to the regularity of their life, for under equally favorable conditions, the ordinary means of cure have failed in many cases. Nor are we allowed to believe that this treatment would have a tendency to produce orchitis, for Dr. Lorey tells us that in his 80 cases he did not meet a single swelled testicle.—*The Doctor.*

Bitter Wine of Iron.—Dr. Charles L. Mitchell, (American Journal of Pharmacy,) says:

"This preparation, so much in demand amongst practitioners at the present time, is, when rightly made, a most elegant tonic and stimulant. As often sold, however, it is of an inky color and taste, and quite repulsive to the patient, Taking advantage of the property which the hydrated sesquioxide of iron possesses, of removing the tannic acid from the different vegetable astringent and tonics, I have succeeded in making a preparation which is handsome, efficient, and pleasant to the taste. The formula is as follows:

Grd. Cinchona Calisaya, 192 grs.; Grd. Gentian Root, 128 grs.; Soluble Citrate Iron, 192 grs.; Sherry Wine, 13 f. ozs.: Brandy, ij. oz.; Alcohol, 1 fl. oz.; Oil Orange, 12 minims.; Sugar, 2 ozs.; Solution Tersulphate of Iron, 2 f. ozs.; Water of Ammonia, q. s.

Dissolve the oil of orange in the alcohol, and mix with the sherry wine and brandy. With this menstrum, percolate the ground drugs, recovering 15 f. ozs. tincture by pouring on water. Dilute the iron solution with twice its bulk of water, and add ammonia until in slight excess. Wash the precipitate until the washings are tasteless, and drain thoroughly. Mix this precipitate with the percolated tincture, and allow to stand, shaking frequently, until a portion filtered off has a light yellow color, and does not blacken with tincture of chloride of iron. Then filter, dissolve the citrate of iron and the sugar, and bring up the measure with a little water to 16 f. ozs.

Each fluid ounce contains 12 grs. cinchona calisaya, 8 grs, gentian root, and 12 grs. of soluble citrate iron.

Salt in Sickness.—Dr. Scudder remarks: "I am satisfied that I have seen patients die from deprivation of common salt during a protracted illness. It is a common impression that the food for the sick should not be seasoned, and whatever *slop* may be given, it is almost innocent of this essential of life. In the milk diet that I recommend in sickness common salt is used freely, the milk being boiled and given hot. And if the patient cannot take the usual quantity in his food, I have it given in his drink. This matter is so important that it cannot be repeated too often, or dwelt upon too long.

"The most marked example of this want of common salt I have ever noticed has been in surgical disease, especially in open wounds. Without a supply of salt the tongue would become broad, pallid, puffy, with a tenacious pasty coat, the secretions arrested, the circulation feeble, the effusion at the point of injury serous, with an unpleasant watery pus, which at last becomes a mere sanies or ichor. A few days of a free allowance would change all this, and the patient get along well."

Dry Earth in Surgery.—Dr. P. S. Conner, of Cincinnati, has been trying the earth treatment, as recommended by Dr. Addinell Hewson, with whom he agrees as to its beneficial effects in the following particulars:

1st. The constant, universal testimony of patients submitted to the earth dressing, enables me to confirm Dr. H.'s statement, that the immediate or primary effect of the direct contact of the earth with the part at least cannot be considered irritating. "Cool and pleasant," is the constant answer of patients when asked how the application feels.

2d. The patients complain less of the pain naturally incident to the injury, than they do under the use of other dressings.

3d. As a deodorizer it can not be too highly praised. In midsummer, the surgical wards of the Good Samaritan Hospital have been entirely freed from noxious odor by a careful attention to the proper application of the earth.

4th. That during the application of the earth dressing there is a marked absence of inflammatory redness around the wound. The granulations are very healthy, and free from any tendency to become large and flabby. Suppuration is certainly less than under the bran or water dressing; thus, it has been seen to diminish more than one-half in the first twenty-four hours after the substitution of the dry earth.

He uses yellow, clayey earth, dried and sifted, which should be made to cover the wound to a depth varying from one-fourth to one, or one and a half inches.—*The Clinic.*

Local Use of Hydrate Chloral in Soft Ulcers and Ulcerated Buboes.—In an article published in the April number of the *Giornale Italiano delle Malattie Venerie*, Dr. De Paoli gives his experience in the local action of chloral hydrate in the above cases, such as was exhibited in the Clinique for Venereal and Skin Diseases of Bologna. Four cases are related, in which large ulcerated buboes were highly benefitted in their last stages by the application of a solution of chloral hydrate (ten parts of chloral to thirty of water). The healing process was remarkably regulated and hastened by the application. The author thinks that in all sores with abundant suppuration and want of tone, chloral is of the greatest use, and that its employment may be beneficially extended, as a slightly exciting and antiseptic agent, to suppurating wounds, and especially gunshot wounds. He states that Professor Gamberini has applied the same solution with marked results to the soft ulcers of prostitutes, especially during the later period of cicatrization, the virulent power and suppuration of the sores being considerably diminished, whilst auto-inoculation of the sores in other parts was not observed in the patients treated. He suggests that chloral hydrate may be a good substitute in certain cases for nitrate of silver and iodoform, and concludes, in summing up his paper, "that it diminishes the virulence of sores, has the advantage of not irritating the inguinal gland, and removes the offensive smell which proceeds from ulcers, especially those of the female genitals."—*London Lancet.*

Treatment of Typhoid Fever.—The following is said (Journal de Medicine) to be the treatment of typhoid fever pursued by M. Peter, Professor Agrege of the hospital de la Charite:

Every other day he gives a glass of Seidlitz water, and morning and evening an emollient injection. The injection he considers to be useful in removing putrid matters. The glass of aperient fluid, without exhausting the patient like a daily purge, also keeps the intestinal canal clear from disintegrated matter. He uses the sulphate of quinine in quantities amounting to from seven to fifteen grains per diem as a febrifuge, increasing the dose in proportion to the intensity of the attack. He places great reliance on alcoholic stimulants, prescribing every day four or five ounces of quinine wine, which is made into a lemonade so as to make from two to three pints of fluid, by which means the vegetable acids are freely and easily administered. These, he thinks, remove the crusts of the tongue, and the symptoms produced by putridity are removed. If the case goes on from bad to worse—if the fever becomes more intense, the temperature increasing, and nervous phenomena supervening—he willingly has recourse to the action of cold, but does not adopt either bath or even cold effusion, which require, he thinks, great circumspection in their application, but contents himself with simply sponging the surface of the body with a sponge dipped in vinegar. Two assistants are required; one lightly passes the wet sponge over the skin, while the second rapidly rubs it with a soft cloth and quickly covers it. He considers one washing per diem to be enough.—*Practitioner.*

Iodine in Incontinence of Urine in Old People.—Dr. Schmidt, of Munsterfield, having witnessed useful effects from the exhibition of iodine in incontinence of urine resulting from paralysis, determined to try it in other cases. An old lady, aged eighty, who had always enjoyed good health, and was very active for her years, was attacked at the age of seventy-six with dysentery, which very much weakened her. From this time the urine passed involuntarily, and for four years she suffered great misery in consequence; from her age her condition was looked upon as incurable. The author gave her one drop of tincture of iodine every hour, and the following day she was able to hold the urine, and she continued the medicine (every two hours one drop) for a fortnight, and with complete success. The discontinuation of the medicine for some time led to a return of the symptoms, which disappeared, however, directly the medicine was resumed. It was continued, therefore, with occasional suspension, for two years, when she died from the effects of a blow.

Another case was an old man, aged seventy-four, who had suffered for six months from the same affection. He was ordered pills, containing each 1-10 grain of iodine. Immediate improvement followed; he was never quite able to do without the remedy. He died eighteen months later, from inflammation of the lungs.—*N. Y. Med. Times.*

Bright's Diease.—Prof. A. L. Loomis, of the University of New York, in a clinical lecture, in speaking of the treatment of this disease, said:

I recommend a remedy which will increase the urinary secretion without stimulating the kidneys. That remedy is digitalis. I am convinced that it does not act as a stimulating diuretic, but that it acts upon the local circulation of the kidney. Clinically, digitalis acts as a diuretic without stimulating the kidneys. Inducing an increased flow of urine in this way we rid the system of its urea much more completely than we can by the skin or bowels, for these are both unnatural ways of eliminating it.

One word with regard to digitalis. We have for many years been taught to believe that while administering this drug, we must guard against its "cumulative effect," so called. All this I have come to disregard; for I have repeatedly administered half-ounce doses of the strong infusion every two hours for 48 hours, and have never seen any unpleasant effects from thus using it. If any benefit is to be received from this drug in this disease, it *must* be administered in large doses. The dose which I usually recommend is ℥ ss of the infusion, and in the acute stage of Bright's disease it may be given every two hours for 24 hours, and then wait a little and watch its effects. If the diuretic effects are not satisfactory, they may be increased by the addition of bitartrate of potassa. Its administration in more moderate doses, say ℥ j three times a day, must be continued for weeks. In those cases in which the exudative matter filling the tubes is the result of a catarrhal inflammation of the tubes, if it can be washed out, nature forms new epithelium for the repair of the tubes, unless the stimulants which have produced the increased secretion of urine are continued. Hence the necessity of watching the effect of the remedy. The external application of dry cups over the kidneys, and following them with poultices, are of service. The digitalis leaves may be used for a poultice after the dry cupping, and thus applied, they will increase the diuretic effect of the drug administered internally. After the use of dry cups, and the congestion and hyperæmia to some extent are relieved, if your uræmic symptoms are still urgent, you may resort to hot-air baths and hydragogue cathartics. This makes the principles for the accomplishment of the first proposition in the management of acute Bright's disease.

And now let us consider the effect of the urea. This is the element that produces the convulsions; perhaps the patient has already had one or more when you are called to see him. What are the means that we have for controlling the effects of urea upon the nervous system? I believe that opium is, of all drugs the best. If called to see a patient who has already had a convulsion, I should not hesitate to throw into his arm 10, 15 or 20 drops of Magendie's solution of morphine, by the use of the hypodermic syringe. It will not kill him, but upon the other hand, I have seen it many times produce a calm, quiet sleep, profuse perspiration, increase the flow of urine, and within a few hours the patient awake to consciousness as the result.

Electrolysis in Cancer.—Dr. Neftel, in *Virchow's Archiv,* recommends a Siemens' battery in the electrolytic treatment of tumors. In moderatly large, hard, and slowly growing tumors, two to four needles are inserted at equal distances into the growth, and are connected with the negative pole, while the positive pole is applied on the skin at some distance. The current is at first weak, and then is quickly increased to thirty-five or forty elements; it is allowed to act at this strength for twenty or thirty minutes, the position of the anode being frequently changed, so that a circle is gradually described around the tumor. The current is then gradually weakened. The application is made every third or fourth day under anæsthesia. the parts of the tumor to which the needles are applied not being the same on each occasion. Generally, four such applications are sufficient; but, to complete the cure, the application for a time of a weaker current (four to eight elements) is necessary. The plate of the cathode is laid on the tumor, and retained there from a quarter to half an hour. This application is not painful, and must be continued for some time after the disappearance of the tumor, in order to prevent the return of the disease. In some cases a clear inodorous alkaline fluid escapes from the needle-punctures after the tumor has become softened. This, according to Neftel, indicates gradual softening and absorption. In very large ulcerated medullary tumors, Dr. Neftel inserts an anode needle into the centre, and four cathode needles close together under the base, changing the position of the latter every twenty minutes, until the whole tumor is undermined. After a week or ten days, it sloughs off, and the weak current is then applied as above described.

Hydrate of Chloral in Labor.—By Dr. A. Pellisier. A Monograph, published by A. Delahaye, Paris, 1873. The author concludes from his experiments on animals and observation at the bedside as follows:

1. Hydrate of chloral exercises no injurious influence whatever upon the health of the mother or child.
2. It interferes in no way with the regularity of uterine contractions, produces sleep and diminishes pain.
3. It is especially appropriate in the natural labors of the primipara, in nervous and irritable women, against cramp, renal troubles, uterine colics.
4. It may be administered by the mouth or by injection at all periods of labor.
5. Obstetrical operations demand absolutely the use of *chloroform.—Med. de Paris,* Oct. 18, 1873.

Phosphate of Lime.—This is regarded, by many, as the best method of giving phosphorus. Ten grains dissolved in one ounce of water, to which a few drops of hydrochloric acid is added, is the dose. It is given to advantage in dyspepsia, chlorosis, tuberculosis, for chronic purulant discharges, to pregnant women, in nervous diseases, etc.

Use of Quinine in Febrile Diseases of Children. — E. Hagenbach, (Jahrb. for Kinderheilk., March, 1872). The author's object in this communication is to prove the favorable action of quinine in reducing the temperature in febrile diseases of children, by which means the disease is rendered a milder one, and in many cases complications of troubles of the nervous system and respiratory organs are prevented. He uses quinine in typhoid fever, and in all the various febrile diseases, when the temperature is continuously high for several days; he combines it with baths between 20 deg. and 24 deg. C., repeated every three hours as long as the temperature remains above 39 deg. C. In pneumonia, he uses baths with older children; with smaller children wrapping in cold sheets is often preferred as less dangerous; at the same time he gives quinine.—Scarlatina, the author treats almost exclusively with baths and quinine. This treatment has also been used with good effect in his later cases of erysipelas. Other diseases enumerated as so treated are acute rheumatism, acute periostitis, coxitis, with high fever, and several other surgical diseases. It is the combined action of the bath and of the quinine that the author lays stress upon. The quinine is given in one large dose, or in two smaller doses, at half-hour intervals, and when the temperature is below 39 deg. C., it is left off. No bad after-effects were noted. The amount to be given varies with the age. For children between one and two years old, from 5 to 16 grains; between three and five years, from 8 to 16 grains; between 6 and 10 years, from 10 to 20 grains; between eleven and fifteen years, from 10 to 30 grains. The largest doses are generally to be preferred. The beneficial effects upon the sensorium, the circulation, subjective symptoms, etc., by baths, cold wet sheets, and quinine, are, beyond doubt, as great in children as in adults, the strength is better kept up and convalesence hastened.

A Means of Allaying Pain in the Use of Vienna Paste.—M. Dr. Lesueus speaks of the severe pain produced by this application and his failure to relieve it or prevent it by adding to the paste a third or fourth of its weight of a salt of morphia. Having occasion recently to make the application upon an individual extremely sensitive to pain, he made the experiment of previously douching the surface with ether by means of Richardson's pulverisator. There was no pain whatever when the paste was now applied, though in this same case, the previous use of morphia was totally without effect in allaying it. On three subsequent occasions he took the same precaution before applying it to the larynx in a case of chronic laryngitis. Here, too, there was complete absence of all pain, whereas a previous application without the douche had been excessively severe.—*Bull. Gen. de Therap.*, Nov. 30th, 1872.—*Clinic.*

Asthma.—Dr. D'Evot says: Twelve grms. of flower of sulphur, with one gramme of tartarized antimony are mixed with honey and powdered gum and divided into sixty pills, of which give one night and morning. Saturate paper in a solution of nitre, one drachm to one ounce of water. Dry and burn one sheet night and morning in bedroom.

Treatment of Psoriasis.—(La France Medicale, Jan. 15, 1873.) Psoriasis is not in itself a serious disease, says M. A. de Montmeja, but it is obstinate, and those who have once been affected by it are very liable to relapses. It is often hereditary, manifesting itself only when the adult period is reached, after which it may be either intermittent or inveterate. In the present state of our therapeutical knowledge, we must not imagine that we can effect a radical cure of psoriasis; we may clear the skin, or hasten the evolution of an attack, but it is impossible to prevent relapses. The treatment is divisible into that by local and general means. The general treatment consists in the administration of mild and frequently repeated aperients, and of arsenical and sulphuretted preparations, as well as of those containing cantharides. M. Hardy prefers small doses of the arseniate of soda to the other preparations of arsenic. M. de Montmeja has obtained considerable success from the employment of two drops of tincture of cantharides in a glass of eau sucree, the dose being increased up to thirty drops per diem. Its use, however, requires extreme care and vigilance. Copaiva is sometimes very useful when given internally. In addition to these means, the waters containing sulphur, of Saint Honore-les-Bains, Bareges, Aix-en-Savoie, may be tried, especially in inveterate cases. The local treatment that is found most beneficial is the application of vapor baths, and either warm alkaline or sulphurous baths, with ointments containing the empyreumatic oils. It is rare for sulphuretted oils to prove of any service, and if mercurial ointments are used, care should be taken when the scabs have fallen off, lest salivation be induced. The oil of Cade, with three parts of lard, is very useful.

The Efficacy and Mode of Employing Collodion in Erysipelas.—M. Broca has again recommended the application of collodion in cases of erysipelas in the following manner: a layer of collodion should be applied round the margin of the erysipelatous blush for a distance of from six to eight centimetres, and also over the affected part. The object of the former is to exercise a circular compression, so as to separate the affected part from the rest of the cutaneous surface. It is necessary to examine these layers once or twice daily, and to repair the fissures which occur. The collodion used must be free from oil. It is rare to see the erysipelas spread after these applications, under which it is in a short time extinguished.

At some large hospitals the ethereal solution of nitrate of silver is much used, and with fairly good results. The objections to it are two-fold: first, on account of its blackening effect, which prevents one seeing if the disease be spreading; and secondly, because of its disfigurement, especially when applied to the face.

Salid Oil an Antidote to Strychnia.—A gentleman writes: "So confident am I of the cure by oil that I think I am only doing my duty by letting it be known that a gill of salid oil poured down the throat, if not too late in the application, is enough for a dog, and ought to be tried on a man."

